# Supplementary material for: Habitual intake of fat and sugar is associated with poorer memory and greater impulsivity in humans
Source: PLoS One. 2023 Aug 24;18(8):e0290308. doi: 10.1371/journal.pone.0290308 (PMC10449134; doi:10.1371/journal.pone.0290308)
Supplement: S1 Table — (DOCX) [file pone.0290308.s001.docx]

**S1 Table. Correlations between the different impulsivity measures in Experiment 1**

|  | 2 | 3 | 4 | 5 | 6 |
| --- | --- | --- | --- | --- | --- |
| 1. BIS11 total | 0.85 *** | 0.77 *** | 0.84 *** | 0.09 | 0.24 * |
| 2. BIS11 attention | - | 0.49 *** | 0.65 *** | 0.14 | 0.29 * |
| 3. BIS11 motor |  |  | 0.37 ** | 0.22 | 0.11 |
| 4. BIS11 non-planning |  |  |  | 0.11 | 0.10 |
| 5. Dickman functional |  |  |  |  | 0.39 ** |
| 6. Dickman dysfunctional |  |  |  |  |  |
