# Supplementary material for: Habitual intake of fat and sugar is associated with poorer memory and greater impulsivity in humans
Source: PLoS One. 2023 Aug 24;18(8):e0290308. doi: 10.1371/journal.pone.0290308 (PMC10449134; doi:10.1371/journal.pone.0290308)
Supplement: S2 Table — Data are coefficients and confidence intervals from each mediation model for each mediation pathway. S8 p<0.05, ** p<0.01, *** p<0.001. (DOCX) [file pone.0290308.s002.docx]

**S2 Table. Outcomes of the mediation analyses from Experiment 1 testing the vicious cycle and trait models of how diet, memory and impulsivity were inter-related.**  Data are coefficients and confidence intervals from each mediation model for each mediation pathway. S8 p<0.05, ** p<0.01, *** p<0.001.

| Impulsivity measure | **Vicious Cycle Model** | | | | |
| --- | --- | --- | --- | --- | --- |
|  | Diet and memory  (path a) | Memory and impulsivity  (path b) | Indirect effect  (path ab) | Diet and impulsivity  (path c’) | Total effect |
| BIStotal | 0.237 (0.087, 0.386)  ** | 0.015 (0.008, 0.021)  *** | 0.006 (0.001, 0.010)  * | 0.003 (0.001, 0.006)  * | 0.009 (0.004, 0.014)  *** |
| BISatt | 0.237 (0.087, 0.386)  ** | 0.018 (0.011, 0.026)  *** | 0.004 (0.001, 0.008)  * | 0.008 (0.002, 0.014)  * | 0.013 (0.006, 0.019)  *** |
| DIdys | 0.237 (0.087, 0.386)  * | 0.069 (0.024, 0.115)  * | 0.016 (0.001, 0.031)  * | 0.028 (-0.007, 0.063) | 0.044 (0.010, 0.079)  * |
| Impulsivity measure | **Trait Model** | | | | |
|  | Impulsivity and diet  (path a) | Diet and memory  (path b) | Indirect effect  (path ab) | Impulsivity and memory  (path c’) | Total effect |
| BIStotal | 11.986 (4.079, 19.893)  * | 0.134 (-0.004, 0.271 | 1.603 (-0.412, 3.619) | 11.478 (6.822, 16.134) | 13.081 (8.376, 13.081)  *** |
| BISatt | 10.796 (4.845,16.748)  ** | 0.115 (-0.032, 0.261) | 1.239 (-0.480, 2.958) | 9.563 (5.337, 13.790)  *** | 10.802 (6.756, 14.848)  *** |
| DIdys | 1.477 (0.388, 2.571)  * | 0.179 (0.037, 0.321)  * | 0.264 (-0.027, 0.556) | 1.297 (0.425, 2.169)  ** | 1.561 (0.649, 2.474)  ** |
