# Supplementary material for: Habitual intake of fat and sugar is associated with poorer memory and greater impulsivity in humans
Source: PLoS One. 2023 Aug 24;18(8):e0290308. doi: 10.1371/journal.pone.0290308 (PMC10449134; doi:10.1371/journal.pone.0290308)
Supplement: S3 Table — EMQ, Everyday Memory Questionnaire: 4MT, total score on the Four Mountains Task: BIS total, total score on the Barratt Impulsiveness Scale: ddt k, k-value from the delay discounting task: DFS, scores on the Dietary Fat and Sugar questionnaire: BMI, body mass index: TFEQR, restraint scale from the Three Factor Eating Questionnaire: TFEQD, disinhibition scale from the Three Factor Eating Questionnaire. (DOCX) [file pone.0290308.s003.docx]

**S3 Table. The main regression models from Experiment 2.** EMQ, Everyday Memory Questionnaire: 4MT, total score on the Four Mountains Task: BIS total, total score on the Barratt Impulsiveness Scale: ddt k, k-value from the delay discounting task: DFS, scores on the Dietary Fat and Sugar questionnaire: BMI, body mass index: TFEQR, restraint scale from the Three Factor Eating Questionnaire: TFEQD, disinhibition scale from the Three Factor Eating Questionnaire.

|  | **Predictor** | **Memory measures** | | **Impulsivity measures** | |
| --- | --- | --- | --- | --- | --- |
|  |  | **EMQ** | **4MT** | **BIS total** | **ddt k** |
| **Step1** | **DFS** | 0.346 (0.200, 0.493)  *** | -0.036 (-0.070, -0.002)  * | 0.004 (0.000, 0.008)  * | 0.000 (0.000, 0.000) |
| **Step 2** | **DFS** | 0.369 (0.218, 0.520)  *** | -0.041 (-0.077, -0.006)  * | 0.005 (0.001, 0.009)  * | 0.000 (0.000, 0.000) |
|  | **BMI** | 0.005 (-0.601, 0.610) | -0.034 (-0.176, 0.107) | 0.005 (-0.012, 0.022) | 0.000 (-0.001, 0.001) |
|  | **Age** | -0.411 (-1.001, 0.179) | -0.055 (-0.193, 0.083) | 0.006 (-0.010, 0.023) | -0.001 (-0.002, 0.000) |
|  | **Sex** | 1.183 (-3.161, 5.527) | -0.389 (-1.406, 0.628) | -0.014 (-0.137, 0.108) | -0.001 (-0.007, 0.006) |
| **Step 3** | **DFS** | 0.317 (0.168, 0.465)  *** | -0.040 (-0.075, -0.006)  * | 0.004 (0.000, 0.008)  * | 0.000 (0.000, 0.000) |
|  | **TFEQR** | -0.389 (-0.782, 0.005) | -0.063 (-0.154, 0.028) | 0.001 (-0.010, 0.012)  * | -0.001 (-0.001, 0.000) |
|  | **TFEQD** | -0.063 (-0.632, 0.506) | -0.059 (-0.191, 0.073) | 0.022 (0.006, 0.012)  ** | 0.001 (0.000, 0.002) |
| **Step 4** | **DFS** | 0.349 (0.201, 0.497)  *** | -0.035 (-0.069, -0.001)  * | 0.004 (0.000, 0.008)  * | 0.000 (0.000, 0.000) |
|  | **Hunger** | -0.013 (-0.082, 0.057) | -0.002 (-0.018, 0.014) | -0.001 (-0.003, 0.001) | 0.000 (0.000, 0.000) |
